# Supplementary material for: Pupillometry and the vigilance decrement: Task‐evoked but not baseline pupil measures reflect declining performance in visual vigilance tasks
Source: Eur J Neurosci. 2022 Jan 18;55(3):778–99. doi: 10.1111/ejn.15585 (PMC9306885; doi:10.1111/ejn.15585)
Supplement: Supplementary file 1 — Figure S1. Joint distribution for horizontal (M = 527, SD = 20) and vertical (M = 379, SD = 26) gaze position of all samples included in the analysis for Experiment 1. Figure S2. Joint distribution for horizontal (M = 518, SD = 10) and vertical (M = 388, SD = 14) gaze position of all samples included in the analysis for Experiment 2. [file EJN-55-778-s001.pdf]

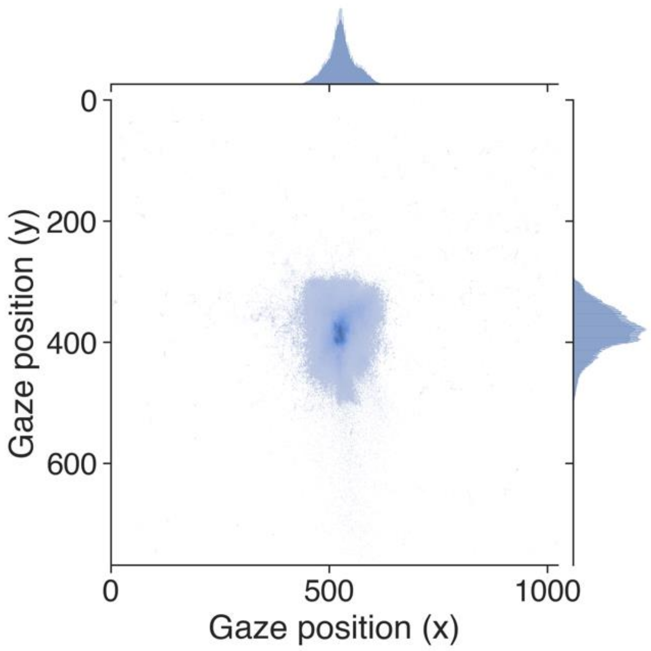

Figure S1. Joint distribution for horizontal (*M* = 527, *SD* = 20) and vertical (*M* = 379, *SD* = 26) gaze position of all samples included in the analysis for Experiment 1.

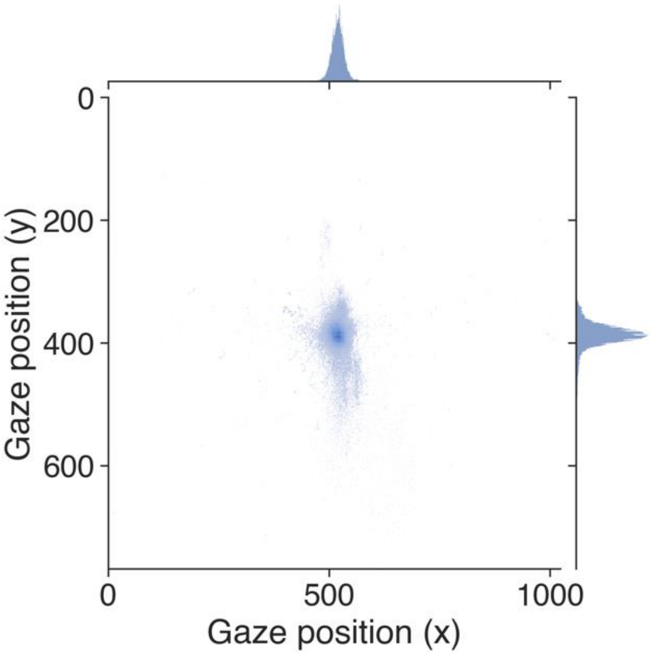

Figure S2. Joint distribution for horizontal (*M* = 518, *SD* = 10) and vertical (*M* = 388, *SD* = 14) gaze position of all samples included in the analysis for Experiment 2.
